# Static or microfluidic? A comparative pilot study of an *in vivo-in vitro* validated human iPSC-derived blood-brain barrier model for neurotoxicity hazard assessment

**DOI:** 10.1016/j.namjnl.2026.100142

**Published:** 2026-10-08

**Authors:** Paul Kurtenbach, Maria Hahn, Mario Thevis, Marc Lamshöft

**Affiliations:** a Bayer AG Division Crop Science, Alfred-Nobel-Strasse 50, Monheim am Rhein, 40789, Germany; b Institute of Biochemistry/Center for Preventive Doping Research, German Sport University Cologne, Am Sportpark Müngersdorf 6, Cologne, 50933, Germany; c European Monitoring Center for Emerging Doping Agents (EuMoCEDA), Bonn, Cologne, Germany

**Keywords:** Blood-brain barrier, Microphysiological systems, Neurotoxicity risk assessment, New approach methodologies, *In vivo-in vitro* validation

## Abstract

Blood-brain barrier (BBB) permeation determines human brain exposure to chemicals and is therefore a prerequisite for neurotoxicity. However, the lack of toxicokinetic BBB models remains a critical uncertainty in regulatory *in vitro* risk assessment, which was highlighted in the new EU roadmap towards phasing out animal testing. Human induced pluripotent stem cell (hiPSC)-derived BBB models combined with clinical positron emission tomography (PET) data provide translational benchmarks for evaluating whether *in vitro* permeability data predict human brain exposure. Microphysiological systems (MPS) may better emulate physiological flow than static transwell® systems. Nonetheless, it remains unclear whether additional complexity introduced by flow improves fitness-for-purpose, reproducibility, and regulatory utility of BBB new approach methodologies (NAMs). Therefore, we directly compared our static hiPSC-derived BBB model, which was previously validated with human clinical PET brain-penetration data, with its microfluidic counterpart to determine whether flow translates into measurable added value for human brain exposure assessment.

The comparative pilot study demonstrated that microfluidic implementation of the static model was technically feasible and maintained key BBB functionality in terms of barrier integrity, efflux capacity, and permeability. However, the MPS displayed reduced *in vivo*-like barrier characteristics and lower brain-penetration predictive performance than the static counterpart within the tested compound set, which is relevant for regulatory neurotoxicity assessment. In addition, the static model offered advantages in standardization, robustness, and throughput that are important for future regulatory implementation. Finally, this study identified technical limitations of the selected microfluidic transwell®-in-chip implementation that may guide the future development of standardized, commercially available BBB systems.

## Highlights

• Implementation of a validated static blood-brain barrier (BBB) in a microfluidic chip.
• Key BBB functionality (barrier integrity, efflux, and permeability) achieved.
• Microphysiological system (MPS) did not improve *in vivo* brain penetration predictivity.
• Technical development potential for future MPS standardization presented.

## Graphical abstract

**Figure:**
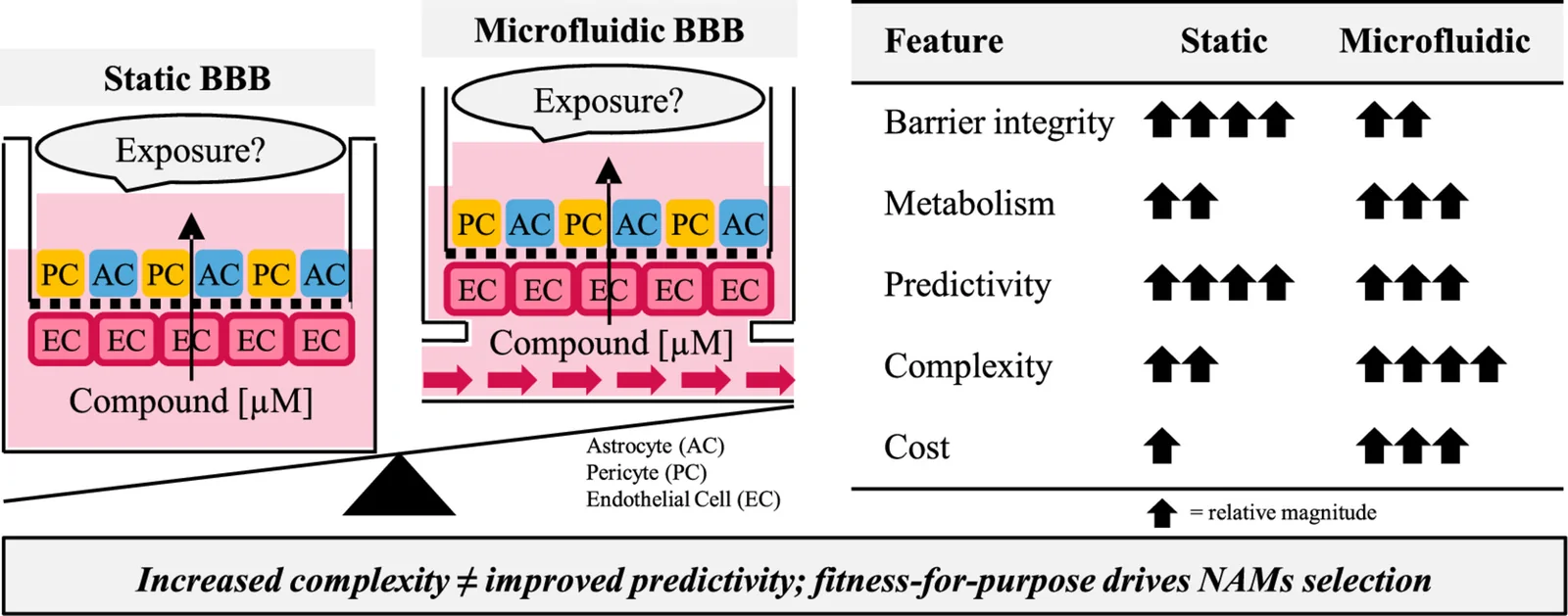

## 1 Introduction

According to the new EU “Roadmap for phasing out animal testing for chemical safety assessments”, the lack of toxicokinetic blood-brain barrier (BBB) models represents a key uncertainty in current *in vitro* neurotoxicity testing (European Commission, 2026a). Toxicokinetic BBB parameters describe the extent to which toxicants cross the BBB and reach the brain, including the resulting exposure concentration, exposure duration and clearance (Illa et al., 2025). Importantly, exposure at the target site is a prerequisite for any compound to trigger a (neuro)toxic effect and is therefore fundamental to downstream neurotoxicity investigations (Leist et al., 2017; Van der Stel et al., 2021). Consequently, BBB models for *in vitro* exposure assessment are critically needed for future animal-free regulatory neurotoxicity testing (European Commission, 2026a; Tal et al., 2024).

For regulatory application, the challenge is to identify model formats that combine physiological relevance with standardization, reproducibility and scalability (Ekert et al., 2020). In line with these principles, static BBB models based on human induced pluripotent stem cell (iPSC)-derived cells were developed, validated with clinical positron emission tomography (PET) and shown to be predictive of human *in vivo* brain penetration, which ultimately determines confidence in a defined regulatory context (Abbott et al., 2022; Delsing et al., 2020; Kurtenbach et al., 2026; Le Roux et al., 2019; Ouedraogo et al., 2025). At the same time, regulators and technology developers have increasingly promoted microfluidic organ-on-a-chip (OoC) systems as advanced model formats that may better emulate human physiology (Escher et al., 2022; European Commission, 2026b; Hartung and Smirnova, 2025). For BBB applications, the key distinguishing feature of these systems is the introduction of flow and associated shear stress (Abbott et al., 2022). Brain microvascular endothelial cells (BMECs), which are largely responsible for BBB integrity, have been reported to respond to shear stress with changes in barrier properties and transporter functionality (Abbott et al., 2022). Nevertheless, due to a lack of direct evidence it has remained unresolved whether the increased complexity associated with a specific microfluidic platform results in a measurable improvement of BBB model performance compared with already validated static systems (Koenig et al., 2022; Yan et al., 2021). Furthermore, implementation of established BBB workflows on microfluidic platforms may introduce additional constraints related to handling, experimental accessibility, throughput, standardization and reproducibility (Yan et al., 2021).

To address this knowledge gap, we transferred our previously published *in vivo-in vitro* validated static human iPSC-derived BBB model (Kurtenbach et al., 2026; Kurtenbach et al., 2025) to a commercially available microfluidic chip platform and directly compared both formats. Beyond demonstrating technical feasibility, we investigated whether microfluidic culture conditions affect metabolic activity, barrier integrity, transporter functionality, and quantitative permeability predictions. Rather than assuming that increased physiological complexity inherently improves model performance, this study systematically evaluated whether the additional complexity introduced by flow and the selected chip platform provides measurable added value for human-relevant brain exposure assessment. As a comparative pilot study, all cell types were derived from a single donor, enabling a controlled comparison of the two culture formats while limiting the generalizability of the findings across donors.

## 2 Materials and methods

### 2.1 Cultivation process of the microfluidic BBB model

HiPSC-derived blood-brain barrier cells (brain microvascular endothelial cells (BMECs), astrocytes and pericytes (APs)), media (BMEC maintenance medium and AP medium), and media supplements were acquired from Fujifilm Cellular Dynamics Inc. (FCDI, Madison, WI, US, catalogue: R1241) as previously described for our static BBB model (Kurtenbach et al., 2026; Kurtenbach et al., 2025). Certificates of analysis for each cell type including cell characterization data are compiled in Supplementary Data File 1. Each cell type has been derived from the same proprietary male donor. Experiments in this study were performed following our previously published static 96-transwell® model set-up process (Kurtenbach et al., 2025). Adaptations to the microfluidic setting were made as described before (Koenig et al., 2022). In the transferred transwell®-in-chip configuration used here, permeability experiments were performed by dosing the basolateral compartment, which was operationally defined as the donor/blood-facing side, while the apical compartment served as the acceptor/brain-facing side. Importantly, the entire microfluidic model set-up and experimental process including permeability experiments was initially run without cells to perform an investigation of non-specific binding of compounds to microfluidic chip material.

Briefly, two days before BMEC seeding (day -2), eight-transwells® strips were cut out vertically out of high-throughput plates (Corning, CLS3392) using a hot scalpel. Next, transwells® were flipped upside down, coated with 50 µL Fibronectin-Collagen IV solution on the basolateral side (4 h, 37 °C and 5 % CO_2_) and stored overnight at 4 °C. On the following day, 96-transwells® were flipped to normal orientation, placed into a receiver plate, and then coated with 0.1 % Gelatin for 30 min at room temperature (RT). APs were seeded in 75 µL AP medium as recommended by the supplier (35 µL 6.36 × 10^5^ astrocytes/mL and 35 µL 1.27 × 10^6^ pericytes/mL). Collagen IV-Fibronectin coating solution was replaced with AP plating medium. After 24 h in the incubator, 96-transwells® were flipped upside down to seed BMECs at 1.859 × 10^6^ BMECs/mL in 50 µL BMEC plating medium onto the basolateral side. In this orientation, cells were incubated to attach for 4 h at 37 °C and 5 % CO_2_ before flipping the 96-transwells® slowly back to normal orientation and filling the basolateral compartment to a total volume of 235 µL. Furthermore, 24 h before the insertion of cells, microfluidic chips (HUMIMIC Chip2®, chip material: cyclic olefin copolymer (CoC), TissUse GmbH, Berlin, Germany) were equilibrated with 352.5 µL BMEC maintenance medium per compartment as recommended by the supplier. On day 1, individual 96-transwells® were cut out of transwell® strips using a hot scalpel and transferred into each compartment of a microfluidic chip using tweezers. Within microfluidic chips, cells in each compartment were cultured in BMEC maintenance medium (352.5 µL basolaterally and 75 µL apically). Next, chips were connected to a HUMIMIC starter (TissUse GmbH, Berlin, Germany), which was operated at a pumping frequency of 1 Hz and 600 mbar pressure, -600 mbar vacuum during the culture and experimental process. After 24 h, barrier integrity was analyzed (Section 2.3) and 24 h-old maintenance medium was exchanged with fresh maintenance medium. On day 3, barrier integrity was assessed again before the 24 h-old medium could be replaced with spiked medium for assaying.

### 2.2 Analysis of carbohydrates

Analogously to our investigation of static BBB carbohydrate metabolism (Kurtenbach et al., 2026), 50 µL aliquots of 24 h-incubated apical and basolateral media were sampled to measure glucose and lactate on days 2 and 3 of the microfluidic BBB cultivation process. Glucose was measured using a GlucCell® Glucose Monitoring System (ESCO Healthcare, Singapore). Each sample was analyzed by applying 3 µL sample volume to one test strip per sample and then operating the hand-held device according to the manufacturer’s instructions. Glucose consumption of quadruplicates was calculated by subtraction of cell sample measurements from blank readings. Lactate was analyzed with a colorimetric kit (Abcam, Cambridge, United Kingdom, catalogue: ab65331) following the quick assay procedure outlined in the manufacturer’s instructions. For blank and cell sample readings, 10 µL blank sample volume and 1 µL cell sample volume was diluted with 40 µL and 49 µL Lactate Assay Buffer, respectively. Absorbance was measured at 450 nm on a microplate reader. Lactate production of quadruplicates was calculated by subtraction of blank measurements from cell sample readings.

### 2.3 Assessment of barrier integrity

Barrier integrity of microfluidic models was investigated in terms of transendothelial electrical resistance (TEER) and sodium fluorescein permeability as published before for static conditions (Kurtenbach et al., 2025). TEER was measured in triplicates on day 2 and day 3, utilizing an EVOM 3 Volt/Ohm meter (World Precision Instruments, Friedberg, Germany) with a STX HTS electrode for 96-transwells® as well as a custom-made 96-transwell® holder (TissUse GmbH, Berlin, Germany). To perform the measurement, 300 µL medium was preloaded into the holder. Next, single 96-transwells® were transferred from their respective chip compartment into the holder using tweezers. Finally, a custom-made holder cap was placed onto the holder, which was designed to accommodate a STX HTS electrode for 96-transwells® in a fixed position to measure TEER. TEER was calculated as the product of electrical resistance and the respective surface area. BBB-models exhibiting TEER ≥ 400 Ω cm^2^ after blank subtraction, which has been described as small molecule-restrictive (Abbott et al., 2022), were considered suitable for permeability testing and *in vivo-in vitro* validation. For TEER validation, sodium fluorescein permeability was investigated as previously described (Koenig et al., 2022; Kurtenbach et al., 2025) with minor adaptations to the microfluidic chip set-up: basolateral medium was replaced with 10 µM sodium fluorescein (Supplementary Data File 2, Table 1) in BMEC maintenance medium. After incubating microfluidic chips for 60 min at 37 °C, 5 % CO_2_, 50 µL were sampled from basolateral and apical chip compartments. In 96-well plates, samples were diluted 1:1 with 50 µL of DPBS. Fluorescence was measured with a SPARK microplate reader (TECAN, Männedorf, Switzerland) with 485/20 nm excitation and 530/20 nm emission. The apparent permeability coefficient *P_app_*, expressed in cm/s, was calculated as previously published (Le Roux et al., 2019):

(1) $$\begin{array}{c} P_{\text{app}}\;=\left(\frac{c_{\text{ac}}}{t\cdot A}\right)∙\left(\frac{V_{\text{ac}}}{c_{\text{dc}}\;}\right) \end{array}$$

**Table 1:** Summary of LC-HRMS parameters and analyte characteristics for analytes and internal standards.

Analyte	Precursor Ion [M+H]^+^[*m/z*]	Quantifier Ion [*m/z*]	Qualifier Ion [*m/z*]	Retention Time (RT) [min]	Normalized Collision Energy (NCE)
Loperamide C_29_H_33_ClN_2_O_2_	477.2303	477.2303	266.1534	7.13	30
Verapamil C_27_H_38_N_2_O_4_	455.2904	303.2090	165.091	6.57	40
Verapamil-d_3_ C_27_H_35_D_3_N_2_O_4_	458.3093	458.3093		6.56	40
Flumazenil C_15_H_14_FN_3_O_3_	304.1092	304.1092	276.0774	6.73	30
Flumazenil-d_5_ C_15_H_9_D_5_FN_3_O_3_	309.1406	309.1406		6.71	30

Where *c_ac_* was the analyte concentration in the acceptor compartment (µM), *t* the duration of the incubation (s, here 3 600 s), *A* the surface area of the respective 96-transwell® membrane (cm^2^), *V_ac_* the volume of the acceptor compartment (mL) and *c_dc_* the applied concentration in the donor compartment (µM). *P_app_* was reported as the average of quadruplicate 96-transwell® model replicates. Barrier integrity was evaluated as sufficiently high when *P_app_* was below 3 × 10^−6^ cm/s (Abbott et al., 2022).

### 2.4 Efflux transport characterization

To compare microfluidic efflux transporter functionality with that of our static BBB model (Kurtenbach et al., 2026), the medium in triplicate basolateral chip compartments was replaced with either fresh medium or fresh medium spiked with 10 µM cyclosporine A (CsA, Supplementary Data File 2, Table 1)). After 60 min at 37 °C, 5 % CO_2_, basolateral medium in CsA-pretreated triplicates, was exchanged for fresh medium spiked with 10 µM CsA and 5 µM verapamil (Supplementary Data File 2, Table 1). Medium of non-pretreated triplicates was replaced with fresh medium spiked with 5 µM verapamil. After 60 min under microfluidic conditions at 37 °C, 5 % CO_2_, entire apical and basolateral compartment volumes were sampled from triplicate microfluidic models as well as cell-free controls. To assess efflux transporter functionality, the unidirectional basolateral efflux ratio (ER) was calculated by dividing the *P_app_* of verapamil with CsA by the *P_app_* of verapamil without inhibitor as outlined before (Fischer et al., 2021):

(2) $$\begin{array}{c} ER_{\text{unidirectional}}=\left(\frac{P_{\text{app}+\text{CsA}}}{P_{\text{appCsA}}}\right) \end{array}$$

### 2.5 Permeability of selected pharmaceutical PET compounds

For permeability characterization in this microfluidic pilot study, a low-permeability compound (loperamide) and a high BBB penetrator (flumazenil) were selected in addition to verapamil as described in other microfluidic studies before (Koenig et al., 2022; Wang et al., 2017). Permeability experiments followed the protocol established for our static BBB model (Kurtenbach et al., 2025) with adaptions to the microfluidic set-up. First, medium in the basolateral compartment was replaced with 352.5 µL fresh medium spiked with 5 µM loperamide, verapamil or flumazenil. Medium in the apical compartment was replaced only with 75 µL fresh medium without compound addition. After microfluidic incubation for 60 min at 37 °C, 5 % CO_2_ entire apical and basolateral compartment volumes were sampled from the apical and basolateral compartments of triplicate models as well as cell-free controls. The incubation time and exposure concentration were selected based on clinical human PET studies used for *in vivo-in vitro* validation with plasma concentrations close to 5 µM and 60 min PET scans (Bauer et al., 2015). In addition, this would maintain consistency with the previously validated static BBB protocol (Kurtenbach et al., 2025), thereby enabling direct comparison of microfluidic and static permeability data against PET-derived human brain-exposure rankings (Kurtenbach et al., 2025). After sampling and cytotoxicity testing, microfluidic chips and 96-transwells® were rinsed apically (75 µL) and basolaterally (352.5 µL) with an acetonitrile/methanol 50:50 (v/v) solution. After 30 min at 37 °C, 5 % CO_2_ the entire rinsing volumes were sampled again.

#### 2.5.1 Sample preparation and analysis

For sample preparation, 5 µL internal standard (verapamil-d_3_, flumazenil-d_5_) was first added to 50 µL sample volume (Supplementary Data File 2, Table 1). As previously described (Kurtenbach et al., 2025), samples underwent protein precipitation using a 50:50 (v/v) mixture of acetonitrile and methanol. After centrifugation for 10 min at 10 000 × *g*, supernatants were transferred to new vials and subsequently evaporated to dryness. Dry samples were reconstituted in an acetonitrile/H_2_O 50:50 (v/v) solution before analysis by liquid chromatography high-resolution mass spectrometry (LC-HRMS/MS). For all analytes, a volume of 5 µL was injected into the LC-HRMS/MS system.

The key LC-HRMS parameters for all analytes are summarized in Table 1. In addition, a comprehensive description of sample analytical procedures is provided in the respective supplementary file (Supplementary Data File 2).

#### 2.5.2 Quantification of BBB penetration

As previously outlined (Kurtenbach et al., 2025) all compounds were quantified using a standard calibration series, and quality control samples prepared in a blank matrix. An individual concentration range was established for each analyte. Method performance including linearity, accuracy, and precision, was characterized based on the analytical requirements issued by the German Society of Toxicological and Forensic Chemistry (GTFCh) (Supplementary Data File 3) (Peters et al., 2009). Raw data were processed for quantification using the Quan Browser in Xcalibur (version 2.2) by Thermo Fisher Scientific. Peak area ratios of analyte and internal standard were plotted against nominal concentrations to generate standard calibration curves. The respective linear functions were then used to calculate the analyte concentrations in unknown samples. For the calculation of recovery rates, the analyte concentration in known apical and basolateral volumes was used to calculate the total analyte amount in apical and basolateral compartments at the beginning and et the end of the permeability experiments. *In vitro P_app_* was calculated for each compound according to the permeability equation described in Section 2.3.

### 2.6 LDH-cytotoxicity

According to our recently published procedure (Kurtenbach et al., 2025) the compounds used in this study were tested for cytotoxic effects on the BBB-cells using the lactate dehydrogenase (LDH)-cytotoxicity assay (Thermo Fisher Scientific, C20301), which was performed immediately after the permeability studies. The assay was performed according to the manufacturer’s instructions with adaptations to the 96-transwell® setting and the microfluidic chip: maximum LDH release was induced by replacing 50 % of the apical and basolateral medium with lysis buffer. After incubation for 45 min at 37 °C, 5 % CO_2_, 50 µL were sampled from both compartments of all samples. Absorbance was measured at 450 and 690 nm using a microplate reader. All sample readings were corrected for background signal (690 nm reading) and blank measurements. Cytotoxicity was calculated for all samples as the percentage of maximum LDH release in apical and basolateral compartments.

## 3 Results

### 3.1 Characterization of barrier integrity and carbohydrate metabolism under microfluidic conditions

The analysis of glucose showed that apically cells consumed nearly 200 nmol on day 2 and day 3 (Fig. 1A). Basolaterally, glucose consumption was close to 500 nmol on day 2 and increased to slightly >600 nmol on day 3 (Fig. 1A). Both levels were not significantly different from the respective static model data (Fig. 1A). Lactate production remained close to 200 nmol apically while basolateral lactate increased non-significantly (Independent samples T-test, p > 0.05) from nearly 740 nmol on day 2 to nearly 820 nmol on day 3 (Fig. 1B). Except for the basolateral compartment on day 3, cells produced significantly less lactate under microfluidic conditions compared to the static model (Fig. 1B). The lactate-to-glucose ratio was close to 1.5 in both compartments on both days, which was also lower than under static conditions (Fig. 1C). Finally, on day 2 and day 3 the concentration of lactate was significantly higher in the apical compartment than in the basolateral compartment, which was similar to observations in the static model (Fig. 1D).

**Fig. 1:**
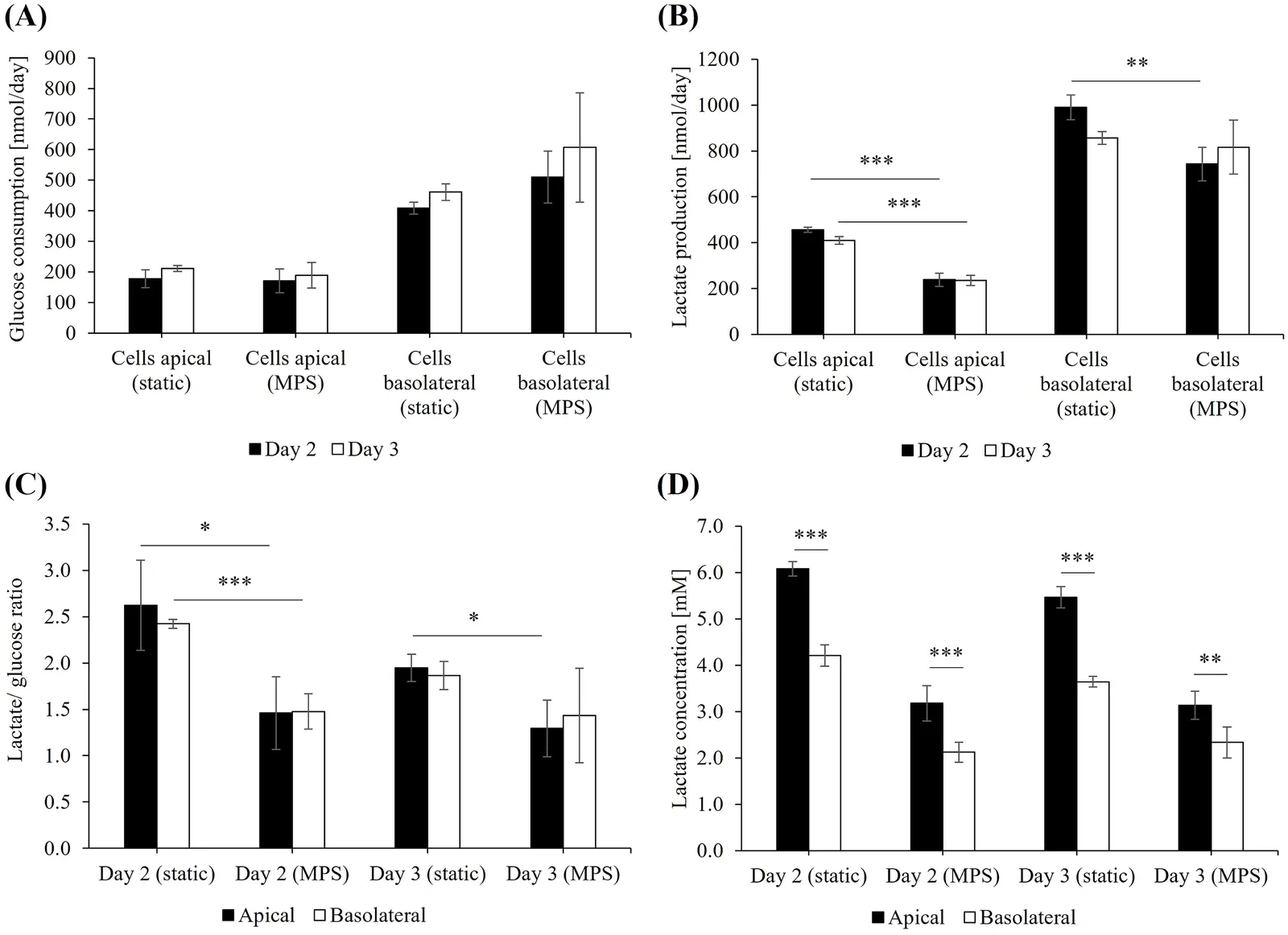
Comparative analysis of carbohydrates under static and microphysiological system (MPS) conditions. Glucose consumption and lactate production in apical and basolateral media were analyzed on days two and three using a GlucCell® system and an Abcam colorimetric kit respectively, calculating values by subtracting blank readings from cell samples. Results were presented as means of four biological replicates: (A) absolute glucose consumption [nmol] per day (and standard deviation) (B) absolute lactate production [nmol] per day (and standard deviation) (C) the ratio of lactate and glucose per compartment (and standard deviation) (D) lactate concentration [mM] (and standard deviation) in each compartment. Statistical significance was calculated using the independent samples T-test (*p < 0.05, **p < 0.01, ***p < 0.001). Static model data are presented as triplicates from day 2 and day 3 after seeding and standard deviation, which we had published before (Kurtenbach et al., 2025). Previously published data were included to facilitate the direct comparison of models.

The restrictive capacity of the microfluidic BBB model was investigated in terms of TEER and paracellular marker permeability. TEER measurements revealed that in the MPS TEER nearly doubled from day 2 (overall average of 428 Ω cm^2^) until day 3 (overall average of 843 Ω cm^2^) (Fig. 2). Triplicate BBB models used for permeability testing displayed average TEER values between 700 and 930 Ω cm^2^. Compared to the static model, TEER values were significantly lower under microfluidic conditions (Fig. 2). Subsequent analysis of sodium fluorescein permeability showed an average *P_app_* of 2.31 × 10^−6^ cm/s in quadruplicate microfluidic models (Supplementary Data File 4). Paracellular marker permeability was therefore significantly higher in the MPS than in the static model (Supplementary Data File 4).

**Fig. 2:**
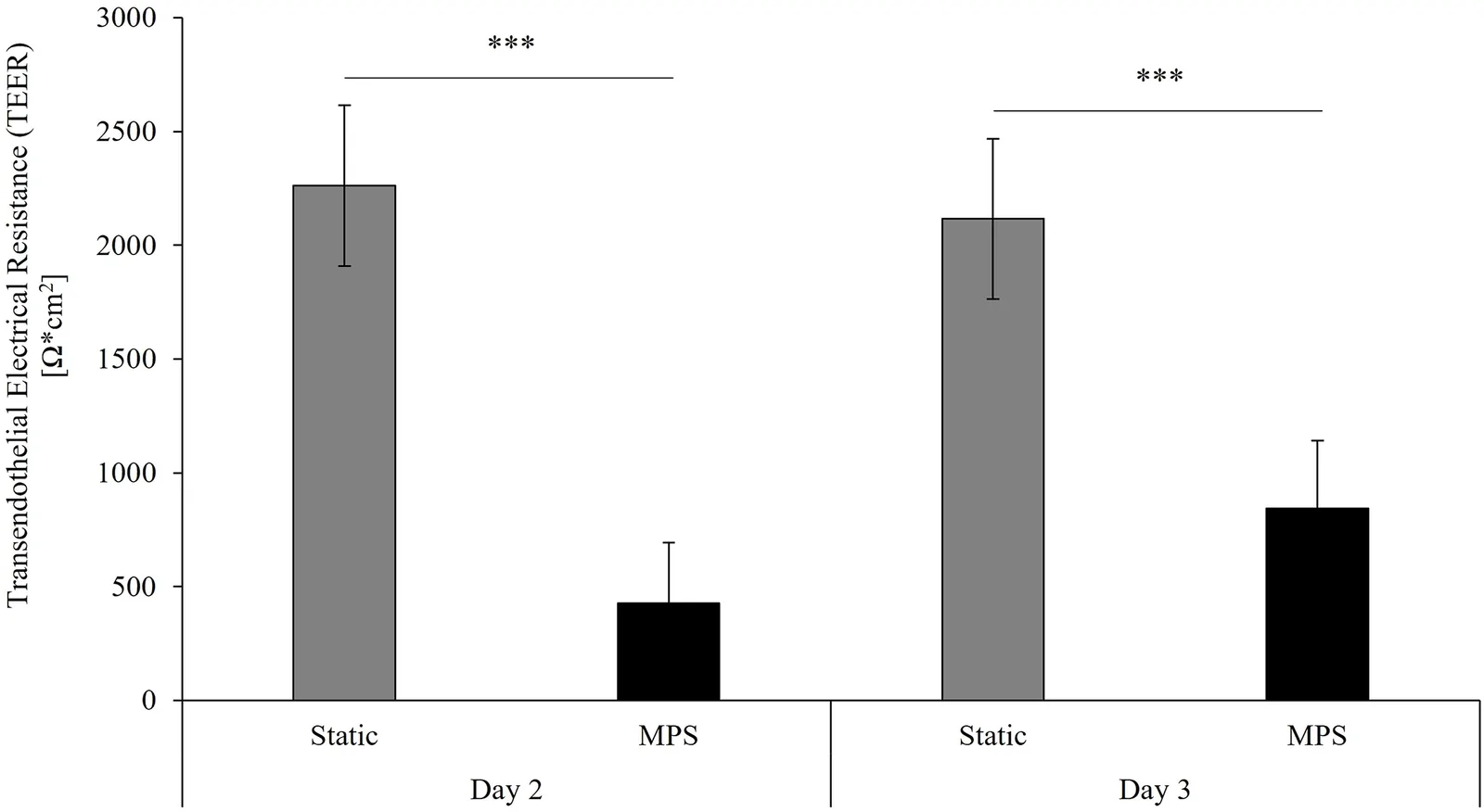
Comparative transendothelial electrical resistance (TEER) of the static and microfluidic hiPSC-derived blood-brain barrier (BBB) model before permeability testing. Average TEER of 18 microfluidic 96-transwells® after subtraction of blank transwells®, which were later used for permeability testing. Measurements of cut-off 96-transwells® were enabled by a custom-made 96-transwells® holder. Before 96-transwells® were transferred from their respective chip compartment into the holder, 300 µL medium were preloaded into the holder. Finally, a custom-made holder cap was placed on top, which was designed to accommodate a STX HTS electrode for 96-transwells®. Black bars (microphysiological system (MPS)) report average TEER values from day 2 and day 3 after seeding and standard deviation. Barrier integrity in terms of TEER was used as an indicator for the optimal day of compound application and permeability testing. Grey bars (static model) report average TEER values from quadruplicates from day 2 and day 3 after seeding and standard deviation, which we had published before (Kurtenbach et al., 2025). Previously published data were included to facilitate the direct comparison of models. Statistical significance was calculated using the independent samples T-test (*p < 0.05, **p < 0.01, ***p < 0.001).

### 3.2 Brain penetration of pharmaceutical PET compounds

The preliminary investigation of non-specific binding of pharmaceuticals to microfluidic chip material showed mean recovery rates of 71 % for loperamide, 90 % verapamil, 82 % verapamil (in combined treatment with CsA) and close to 100 % flumazenil (Supplementary Data File 5) after 60 min of microfluidic incubation. A comparison of these cell-free recovery rates with the recoveries after the final experiments with cells did not reveal significant differences (Supplementary Data File 5).

Using verapamil as a P-gp substrate for efflux transporter functionality analysis, microfluidic BBB models exhibited an *in vitro P_app_* of 18 × 10^−6^ cm/s (Fig. 3). P-gp inhibition with CsA led to a significant increase in substrate permeability (25 × 10^−6^ cm/s), which equated to a basolateral unidirectional ER of 1.35 (Fig. 3). Under static conditions, this ratio was more than twice as high (ER = 2.9), resulting from a lower *in vitro P_app_* of 13 × 10^−6^ cm/s without P-gp inhibition and a higher *P_app_* of 38 × 10^−6^ cm/s after P-gp inhibition.

**Fig. 3:**
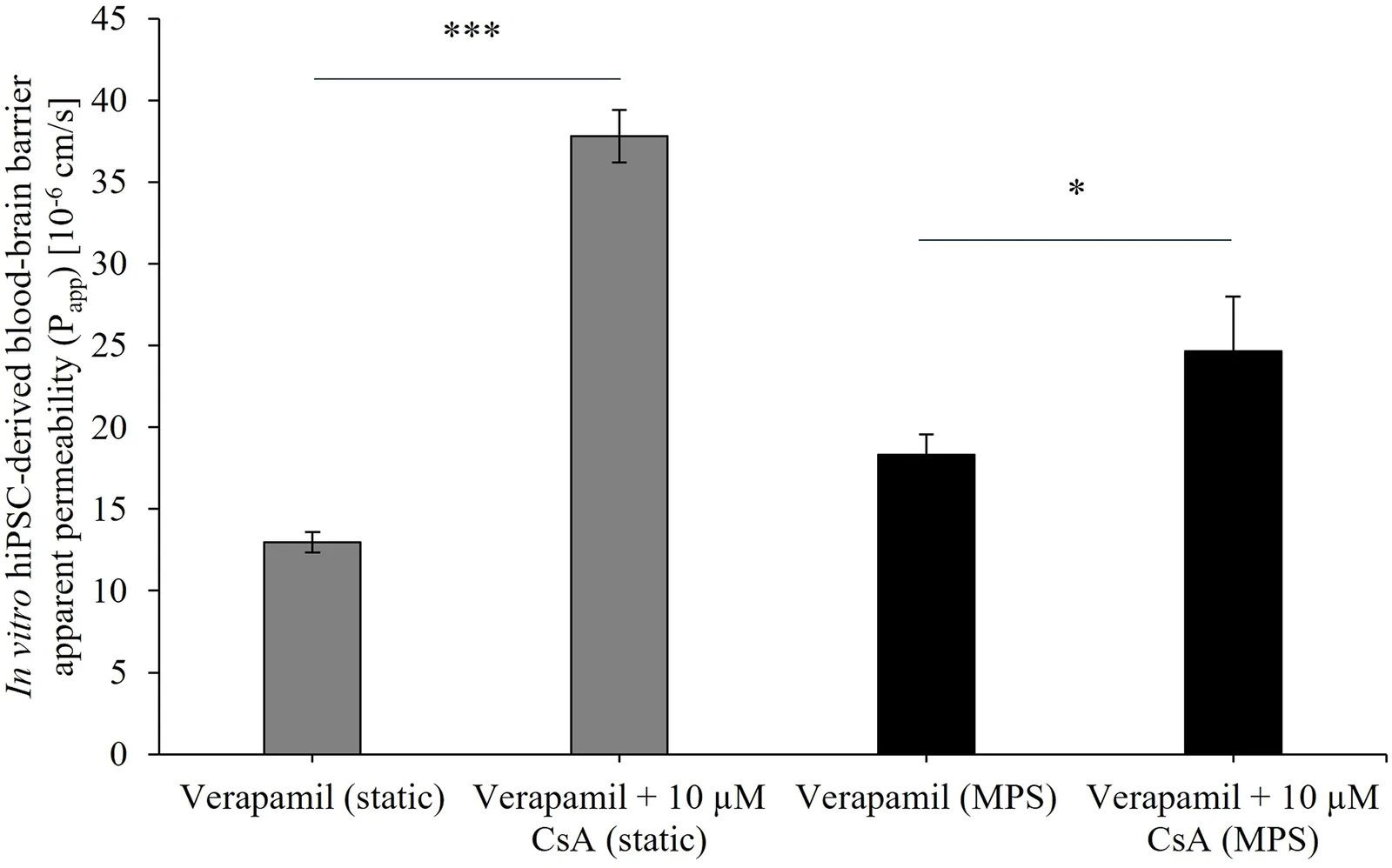
Comparative efflux transporter functionality under static and microphysiological system (MPS) conditions. P-glycoprotein (P-gp) functionality was assessed by measuring the permeability of verapamil across microfluidic BBB models with and without the P-gp inhibitor CsA. Triplicate BBB models were pretreated with 10 µM CsA or fresh medium (60 min at 37 °C, 5 % CO_2_), then exposed to 5 µM verapamil with or without 10 µM CsA (60 min at 37 °C, 5 % CO_2_), and compound concentrations were analyzed by liquid chromatography-high resolution mass spectrometry LC-HRMS. Bars represent mean apparent permeability (*P_app_*) of three biological replicates per compound (and standard deviation). Data from static models (grey bars) have been included for comparative purposes from our recent BBB publication (Kurtenbach et al., 2026), which was foundational for the efflux transporter characterization under microfluidic conditions in this study. Statistical significance was calculated using the independent samples T-test (*p < 0.05, **p < 0.01, ***p < 0.001).

The examination of *in vitro P_app_* of pharmaceutical PET tracers revealed significantly increased penetration of the BBB under microfluidic conditions by loperamide (*P_app_* > 10 × 10^−6^ cm/s) and verapamil (*P_app_* > 15 × 10^−6^ cm/s) compared to their static counterparts (Fig. 4). High permeability of flumazenil (*P_app_* > 30 × 10^−6^ cm/s) remained unchanged after exposure to flow (Fig. 4). Compared to our recent BBB publication of static models (Kurtenbach et al., 2025), the microfluidic model showed a smaller dynamic range of increasing permeabilities of tested compounds (Fig. 4).

**Fig. 4:**
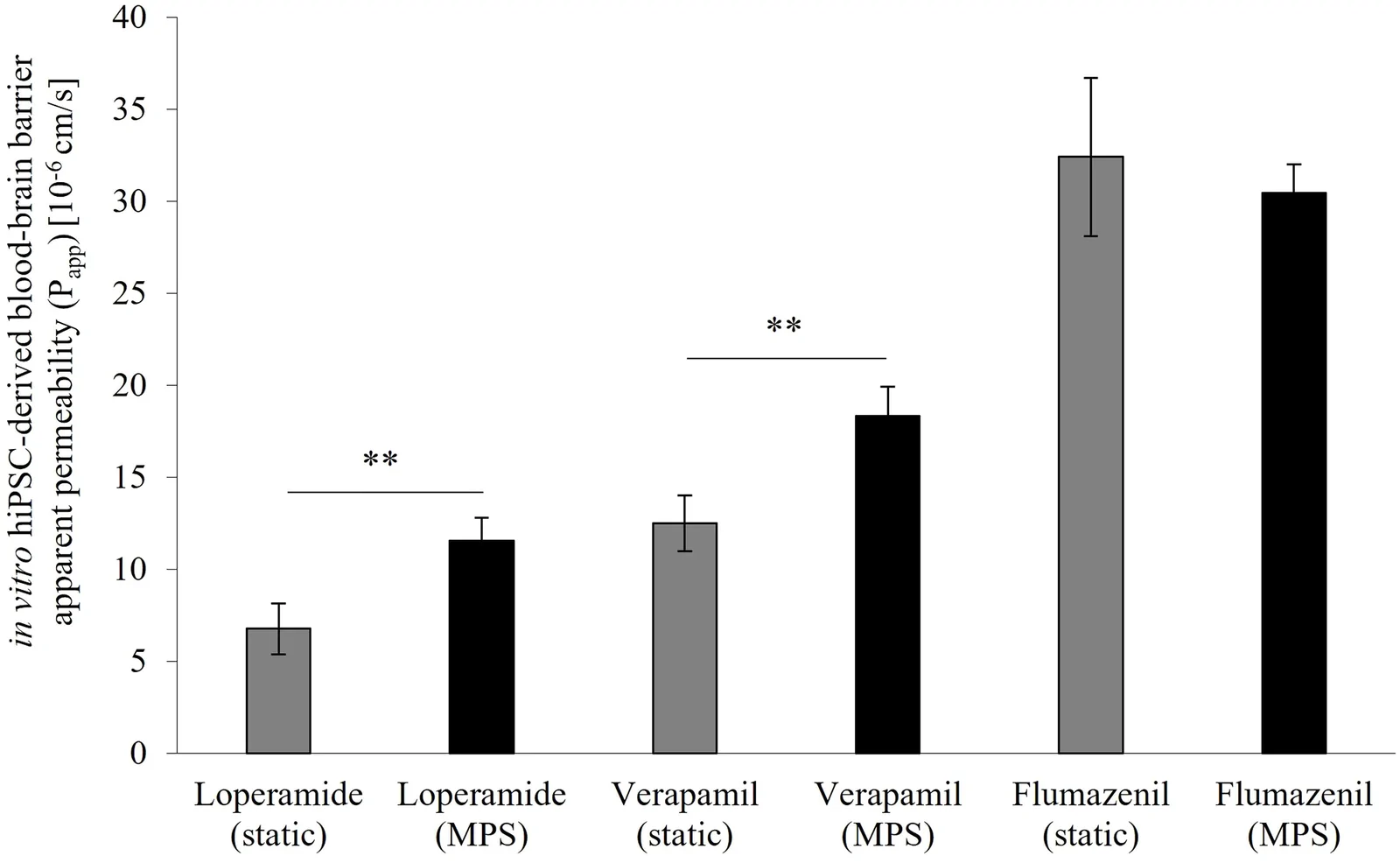
Comparative permeability of pharmaceutical positron emission tomography (PET) compounds under static and microphysiological system (MPS) conditions. *In vitro* apparent permeability (*P_app_*) of compounds was investigated by replacement of medium in the basolateral compartment with fresh medium spiked with 5 µM loperamide, verapamil or flumazenil. After 60 min at 37 °C, 5 % CO_2_, entire compartment volumes were sampled from apical and basolateral compartments of 96-transwells® in microfluidic chips. The compound concentration was analyzed by liquid chromatography-high resolution mass spectrometry (LC-HRMS/MS). Bars represent mean *P_app_* of three biological replicates per compound (and standard deviation). Data from static models (grey bars) have been included for comparative purposes from our first BBB publication (Kurtenbach et al., 2025), which was foundational for the microfluidic model set up and permeability testing in this study. Statistical significance was calculated using the independent samples T-test (*p < 0.05, **p < 0.01, ***p < 0.001).

Cytotoxicity analysis after compound incubation in the permeability experiments showed no significant increase in LDH release for pharmaceutical treatments compared to the corresponding medium control (Fig. 5). Baseline cytotoxicity in medium controls of the microfluidic model remained below 20 % relative to lysed cells and was not significantly different to baseline cytotoxicity in the static model (Fig. 5).

**Fig. 5:**
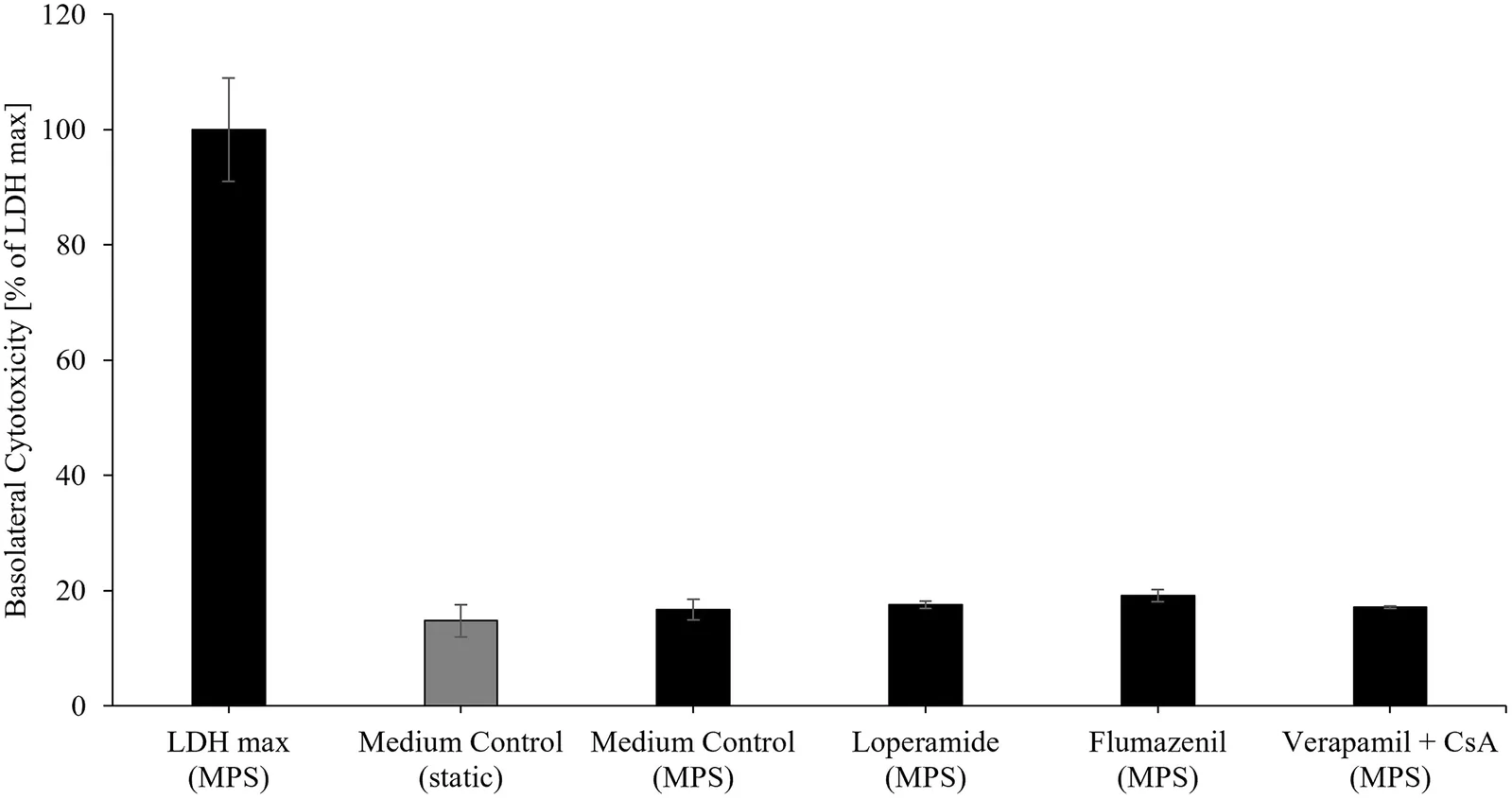
Cytotoxicity of blood-brain barrier (BBB) models and pharmaceuticals.To achieve maximum lactate dehydrogenase (LDH max) release, 50 % of apical and basolateral volumes were replaced with lysis buffer. After incubation under microfluidic conditions for 45 min at 37 °C and 5 % CO_2_, absorbance of medium samples was measured at 450 nm and corrected for background signal (690 nm) and blank measurements. Mean cytotoxicity (and standard deviation) of three biological replicates was reported as the percentage of maximum LDH release in the basolateral compartment. An independent samples T-test was utilized to test statistical significance for each compound as well as base level cytotoxicity under static conditions (grey bar, six replicates, Kurtenbach et al., 2025) against the medium control under microphysiological system (MPS) conditions (*p < 0.05, **p < 0.01, ***p < 0.001) but did not reveal significant differences of pharmaceutical treatments or between models. Previously published static data were included to facilitate the direct comparison of static and microfluidic models.

## 4 Discussion

The first goal of this study was to transfer our recently published *in vivo-in vitro* validated static hiPSC-derived BBB model and permeability testing method (Kurtenbach et al., 2026; Kurtenbach et al., 2025) into a commercially available microfluidic chip platform. Secondly, the direct comparison of static and microfluidic characterization parameters should allow for a systematic evaluation whether the additional complexity introduced by flow and the selected chip platform provide measurable added value compared to the static BBB NAM for human brain exposure assessment.

The analysis of glucose showed constant glucose consumption and lactate production under microfluidic conditions on day 2 and day 3 (Fig. 1A,B). Carbohydrate metabolism therefore indicated constant tissue homeostasis within the microphysiological system as opposed to either elevated cell growth or death, which would impact the overall cell count and thus, metabolism parameters. This general observation was supported by the endpoint cytotoxicity assay (Fig. 5), which did not indicate overt cytotoxicity under the tested conditions (Jablonska et al., 2021; Liu et al., 2018; OECD, 2021). Consequently, the technical handling process of transferring static models into the chip and exposure to flow-associated shear stress did not induce excessive cytotoxicity or major metabolic instability under the tested conditions.

In contrast to static observations (Kurtenbach et al., 2026) cells produced less lactate (Fig. 1B), while consuming similar levels of glucose (Fig. 1A), which led to a comparatively reduced lactate-to-glucose ratio of 1.5 (Fig. 1C). This observation is in line with previous findings, which showed that under microfluidic conditions, endothelial cells tend to rely more on oxidative phosphorylation than on glycolysis (Cucullo et al., 2007; Koenig et al., 2022). For the study presented here, the reduced lactate-to-glucose ratio in the MPS may indicate a partial shift towards less glycolytic metabolism under microfluidic conditions and thus, an improved represantion of the *in vivo* situation compared to our static model. However, the compartment-specific lactate accumulation in the apical acceptor compartment (Fig. 1D) remained inconsistent with a fully physiological brain-side metabolic environment. Considering the presence of astrocytes, which have been shown to be the most glycolytic brain cells and the absence of heavy lactate-reliant neurons in this study, these findings are plausible (Matthiesen et al., 2022). As concluded by others before, a more complex “brain” side, which includes neurons in microfluidic models of the BBB could be a physiologically-relevant development in the future (Koenig et al., 2022). In addition, inclusion of more donors as well as representation of male and female cell resources would be beneficial for future research to foster generalizability of results and accounting for donor effects, which are currently still limiting factors (Seo et al., 2020).

Barrier integrity examinations revealed average TEER values above 700 Ω cm^2^ with minimum benchmark criterion set at 400 Ω cm^2^ for individual transwells® (Fig. 2). Consequently, microfluidic models did not achieve *in vivo*-like properties (TEER > 1 000 Ω cm^2^) as frequently shown for static BBB models (Abbott et al., 2022; Kurtenbach et al., 2026; Stebbins et al., 2016) and some microfluidic models (Fengler et al., 2022; Wang et al., 2017). Nonetheless, TEER ≥ 400 Ω cm^2^ has been described as small molecule-restrictive (Abbott et al., 2022), while TEER ≥ 150 Ω cm^2^ was still suitable for permeability testing (Koenig et al., 2022; Le Roux et al., 2019), which was the goal of this study. Subsequent investigation of sodium fluorescein permeability confirmed the TEER analysis with *P_app_* being close to the benchmark set for *in vivo-*like barrier integrity of 2 × 10^−6^ cm/s (Stebbins et al., 2016) (Supplementary Data File 4). Again, these findings are consistent with relevant literature, which has found fluorescein *P_app_* <1 × 10^−6^ cm/s when TEER was well above 1 000 Ω cm^2^ (Fengler et al., 2022; Wang et al., 2017) and *P_app_* >5 × 10^−6^ cm/s for leaky models such as the BBB cell line hCMEC/D3 (TEER < 100 Ω cm^2^) (Abbott et al., 2022; Balzer et al., 2022; Kurtenbach et al., 2025). Comparing available cell culture material used for the hiPSC-derived model under static (Kurtenbach et al., 2026; Kurtenbach et al., 2025) and microfluidic conditions, could provide some explanations for the lower barrier integrity within the MPS: first, 96-transwells® are only available in high-throughput screening (HTS) plates, which has led to protocols, where transwells® have to be cut out with a hot scalpel (Koenig et al., 2022). Second, cut-out 96-transwells® are also not designed to stand alone as they lack respective feet or hanging features, which means that the outer BMEC cell layer is in direct contact to a ring-shaped lip within the MPS. Third, the static model in HTS plates feature a side channel next to the apical compartment, which leads to less handling of transwells® and impact on cell layers. Given that barrier integrity parameters such as TEER are very sensitive to external physical and technical factors (Vigh et al., 2021) the available transwell® infrastructure had an inevitable impact on the desired direct comparison of a static and a microfluidic setting. Importantly, this study was not intended to evaluate microfluidic BBB concepts in general. Rather, it aimed to determine whether implementation of a validated static BBB NAM on a commercially available microfluidic platform improves model performance sufficiently to justify the increased experimental complexity. From a barrier integrity perspective, the specific transwell®-in-chip implementation presented here did not outperform our validated static model (Kurtenbach et al., 2026; Kurtenbach et al., 2025) under the conditions tested, although it still met accepted standards for permeability testing (Koenig et al., 2022; Le Roux et al., 2019). Besides, this study identified several technical features of standardized, commercially available material, which could be addressed to improve usability and performance of future microfluidic in vitro BBB systems.

To further compare functional capabilities of the microfluidic BBB model, the unidirectional basolateral permeability of P-gp substrate verapamil with P-gp inhibitor CsA was examined as recently published under static conditions (Kurtenbach et al., 2026). Here, a statistically significant increase in brain penetration upon inhibition was found (Fig. 3), which revealed a similar efflux ratio to previous investigations with vinblastine and CsA (ER = 1.5) (Le Roux et al., 2019). Investigations of other static hiPSC-derived BBB models did show efflux functionality of rhodamine 123 but no significant efflux of several pharmaceutical compounds including verapamil (Delsing et al., 2018). Clinically, however, inhibition of *(R)*-[^11^C]verapamil with tariquidar resulted in a 3.5-fold increase in human *in vivo* brain penetration rate (*K*_1_ = 0.043 mL cm^−3^ min^−1^ without inhibitor, *K*_1_ > 0.149 mL cm^−3^ min^−1^ with inhibitor) (Bauer et al., 2015). In addition, by means of a rat BBB model, *K*_1_ of *(R)*-[^11^C]verapamil increased >7 times upon P-gp inhibition with tariquidar (Kuntner et al., 2010). The direct comparison to our recently validated static model (ER = 2.9) (Kurtenbach et al., 2026) revealed less pronounced efflux functionality in the MPS (Fig. 3). Thus, although the microfluidic BBB model retained P-gp-related efflux as a core BBB function, its markedly lower efflux ratio indicates reduced *in vivo*-like transporter performance compared with the validated static counterpart. Nevertheless, this observation remains limited to P-gp-related efflux of verapamil and inhibition with CsA. For future research, it would be desirable to test more efflux substrates, inhibitors and efflux transporters (such as Breast Cancer Resistance Proteins (BCRP)) for IVIV-comparison to further characterize the predictive capacity of the *in vivo* state.

Microfluidic permeability investigations of three pharmaceutical PET tracers confirmed recently published findings for the static model (Kurtenbach et al., 2025) with loperamide to be the weakest brain penetrator, followed by verapamil and flumazenil as the most permeable tested compound (Fig. 4). This order of increasing permeability was also found in previous investigations of PET tracers (Le Roux et al., 2019). Nevertheless, after transferring into the MPS, the permeability of well-known P-gp substrates Loperamide (*P_app static_* <7 × 10^−6^ cm/s, *P_app MPS_* <12 × 10^−6^ cm/s) and Verapamil (*P_app static_* <13 × 10^−6^ cm/s, *P_app MPS_* <18 × 10^−6^ cm/s) increased significantly, while Flumazenil stayed at the same level (Fig. 4). This indicates that the microfluidic model exhibited less *in vivo*-like restrictive properties than the *in vivo-in vitro* validated static model, which showed very limited permeability to both efflux substrates and high correlation to the respective human clinical data (Kurtenbach et al., 2025). Moreover, increasing permeabilities of low-permeability compounds decrease the dynamic range for prediction of *in vivo* brain penetration, which suggests a lower capacity to discriminate CNS from non-CNS compounds (Abbott et al., 2022; Fengler et al., 2022; Kurtenbach et al., 2026; Le Roux et al., 2019). For regulatory neurotoxicity hazard assessment, reduced separation between low- and high-penetrating compounds may increase uncertainty in prioritization decisions, particularly when BBB penetration is used as an exposure gate before downstream neurotoxicity testing. In sum, the microfluidic BBB model presented here displayed important permeability functionalities as other microfluidic models before (Koenig et al., 2022). However, a direct comparison revealed that it did not achieve the resolution of our less complex static alternative (Kurtenbach et al., 2026) or static models presented by others before (Le Roux et al., 2019), which has been shown to be related to highly restrictive barrier properties (e.g., paracellular marker *P_app_* <2 × 10^−6^ cm/s, TEER > 1 000 Ω cm^2^) (Abbott et al., 2022). Our conclusions are limited to the direct comparative conditions of this study, which utilized standardized, commercially available material to foster comparability and to ultimately facilitate the potential integration into regulatory testing schemes for neurotoxicity hazard assessment. These findings support a fitness-for-purpose view of BBB NAM development, in which qualification should be driven by context-of-use performance metrics such as human brain exposure predictivity, discriminatory capacity, robustness, throughput, operational feasibility, and regulatory usability, rather than by model complexity alone. Moreover, the results are in line with previous findings, which indicated that for BBB permeability studies, flow-induced shear stress is not an indispensable design paradigm (Stebbins et al., 2016; Wang et al., 2017).

## 5 Conclusion

This study aimed to systematically evaluate whether the additional complexity introduced by a microfluidic platform provides measurable added value compared with our recently validated static BBB NAM for human brain penetration assessment. Technical feasibility of the MPS transfer was demonstrated by stable carbohydrate metabolism and retained functional BBB properties in terms of barrier integrity, P-gp-related efflux functionality, and a permeability-based compound ranking. However, the systematic comparison also revealed that the microfluidic BBB model showed less *in vivo*-like functional performance compared with the static counterpart. Within the scope of our comparative pilot study, the static BBB model exhibited higher discriminatory resolution for human brain penetration within the tested compound set, which is relevant for future regulatory evaluation, qualification efforts and implementation. These findings highlight that reduced model complexity can be advantageous within a defined context of use when it improves robustness, throughput, and interpretability without compromising the intended predictive purpose. Finally, this study identified technical constraints of the selected microfluidic transwell®-in-chip implementation that may guide future improvements of standardized, commercially available BBB systems.

## Ethics declaration

Written informed consent to take part in the study and to publish the article has been obtained from all participants or their legal representatives. The privacy rights of participants have been observed.

This study included organ or tissue donors. This study includes human biological material and consent was obtained by donors, or their next of kin or legal representatives, for use in this study and for publication of the article. The samples used in this research were not sourced from executed prisoners or prisoners of conscience.

This study was performed in compliance with relevant laws, regulatory frameworks and guidelines where the research took place. Ethics committee approval was not required under relevant laws and institutional guidelines. No additional ethics approval was required for this study. Experiments were performed using commercially available, previously established human iPSC-derived BBB cells from FUJIFILM Cellular Dynamics Inc.; no human participants, human sample collection, animal experiments, or de novo stem-cell derivation were involved. All work was conducted in accordance with applicable institutional biosafety and laboratory guidelines.

## CRediT authorship contribution statement

**Paul Kurtenbach:** Writing – original draft, Visualization, Validation, Project administration, Methodology, Investigation, Formal analysis, Data curation, Conceptualization. **Maria Hahn:** Writing – review & editing, Validation, Supervision, Methodology. **Mario Thevis:** Writing – original draft, Supervision, Resources, Funding acquisition. **Marc Lamshöft:** Writing – review & editing, Supervision, Resources, Project administration, Methodology, Funding acquisition.

## Declaration of competing interest

The authors declare the following financial interests/personal relationships which may be considered as potential competing interests:

Paul Kurtenbach reports financial support was provided by Bayer AG. Maria Hahn reports financial support was provided by Bayer AG. Marc Lamshoeft reports financial support was provided by Bayer AG. Paul Kurtenbach reports a relationship with Bayer AG that includes: employment. Maria Hahn reports a relationship with Bayer AG that includes: employment. Marc Lamshoeft reports a relationship with Bayer AG that includes: employment. If there are other authors, they declare that they have no known competing financial interests or personal relationships that could have appeared to influence the work reported in this paper.

## Data Availability

Data is contained within the article or supplementary material.
