# Supplementary material for: Static or microfluidic? A comparative pilot study of an in vivo-in vitro validated human iPSC-derived blood-brain barrier model for neurotoxicity hazard assessment
Source: NAM J. 2026 Oct 8;2:100142. doi: 10.1016/j.namjnl.2026.100142 (PMC13683853; doi:10.1016/j.namjnl.2026.100142)
Supplement: Supplementary file 1 [file mmc3.pdf]

FUJIFILM Cellular Dynamics, Inc.
525 Science Drive
Madison, WI 53711 USA

Toll Free in US (877) 320-6688 / (608) 310-5100 T
(608) 310-5101 F
fcdi-support@fujifilm.com E
www.fujifilmcdi.com W

Certificate of Analysis

**Product Name:**	iCell® BMEC	**Lot Number:**	107456
**Catalog Number:**	C1239	**Unit Size:**	≥ 3 x 10^6 cells/vial
**Donor:**	01279	**Use By:**	12 months from date of shipment
**Phenotype:**	AHN		
		**Storage Conditions:**	Vapor Phase LN_2

This product is subject to use restrictions, which can be found in FUJIFILM Cellular Dynamics, Inc. Standard Terms and Conditions accessible at https://fujifilmcdi.com/assets/tnc/standard.pdf. Failure to adhere to these use restrictions, among other things, will void the limited warranty applicable to this product.

For further assistance, contact technical support (fcdi-support@fujifilm.com).

Test Parameter	Result
**Viable Cells**	3.7x10^6 cells at thaw by trypan blue exclusion
**Purity**	5 days post-thaw by flow cytometry
	99% GLUT1
	98% TFRC
	81% CD98
	80% MRP1
	96% PGP
**Viability**	63% at thaw by trypan blue exclusion
**Identity Confirmation**	Match by SNP genotyping
**Mycoplasma**	Pass by PCR Assay
**Sterility**	Pass per 21 CFR 610.12
**Barrier Function/Transepithelial Electrical Resistance (TEER)**	>1500 Ohms.cm^2

iPSC Line Authentication Statement

All FUJIFILM Cellular Dynamics, Inc. (FCDI) products are manufactured from tissue-derived induced pluripotent stem cell (iPSC) lines that were produced by FCDI. Each tissue sample received by FCDI (an Original Sample) is assigned a unique 5-digit identification code (ID). FCDI uses SNP analysis on genomic DNA (gDNA) isolated from the Original Sample to establish a DNA fingerprint for each ID.

The iPSC line and iPSC-derived differentiated cell type produced are identified by the Original Sample's ID and DNA fingerprinted to confirm identity with that Original Sample.

In the case a gene-edited iPSC is produced from a particular Original Sample, it is identified by the Original Sample's ID and confirmed using the DNA fingerprint. The gene-editing of the iPSC is confirmed using sequence analysis of the gene-edited region.

This lot passes all quality control specifications.

Quality Assurance Approval: Milena R Havorka Date: 25 Sep 2024

For Research Use Only. Not for use as a therapeutic or diagnostic.

© 2019 FUJIFILM Cellular Dynamics, Inc.

Rev 01

FUJIFILM Cellular Dynamics

FUJIFILM Cellular Dynamics, Inc.
525 Science Drive
Madison, WI 53711 USA

Toll Free in US (877) 320-6688 / (608) 310-5100 **T**
(608) 310-5101 **F**
fcdi-support@fujifilm.com **E**
www.fujifilmcdi.com **W**

Certificate of Analysis

**Product Name:**	iCell® Pericytes	**Lot Number:**	107261
**Catalog Number:**	C1241	**Unit Size**	≥1.5x10⁶ cells/vial
**Donor ID:**	01279	**Use By:**	12 months from date of shipment
**Phenotype:**	AHN	**Storage Conditions:**	Vapor Phase LN_2

This product is subject to use restrictions which can be found in FUJIFILM Cellular Dynamics, Inc. Standard Terms and Conditions accessible at https://fujifilmcdi.com/assets/tnc/standard.pdf. Failure to adhere to these use restrictions, among other things, will void the limited warranty applicable to this product.

For further assistance, contact technical support (fcdi-support@fujifilm.com).

Test Parameter	Result
**Viable Cells**	$1.7x10^6$ cells at thaw by Trypan blue exclusion
**Purity,**	5 days post-thaw by Flow Cytometry
	96% PDGFR-β (CD140b)
	85% NG2
	94% CD13
	>99% Desmin
	99% αSMA
**Viability**	82% at thaw by Trypan blue exclusion
**Identity Confirmation**	Match by SNP genotyping
**Mycoplasma**	Pass by PCR Assay
**Sterility**	Pass per 21 CFR 610.12

iPSC Line Authentication Statement

All FUJIFILM Cellular Dynamics, Inc. (FCDI) products are manufactured from tissue-derived induced pluripotent stem cell (iPSC) lines that were produced by FCDI. Each tissue sample received by FCDI (an Original Sample) is assigned a unique 5-digit identification code (ID). FCDI uses SNP analysis on genomic DNA (gDNA) isolated from the Original Sample to establish a DNA fingerprint for each ID.

The iPSC line and iPSC-derived differentiated cell type produced are identified by the Original Sample's ID and DNA fingerprinted to confirm identity with that Original Sample.

In the case a gene-edited iPSC is produced from a particular Original Sample, it is identified by the Original Sample's ID and confirmed using the DNA fingerprint. The gene-editing of the iPSC is confirmed using sequence analysis of the gene-edited region.

This lot passes all quality control specifications.

Quality Assurance Approval: ________________ Date: 16 May 2024

For Research Use Only. Not for use as a therapeutic or diagnostic. TEMP-00034.01 Effective Date:30 Sep 2019
© 2019 FUJIFILM Cellular Dynamics, Inc.
Rev 00

FUJIFILM
Cellular Dynamics

FUJIFILM Cellular Dynamics, Inc.
525 Science Drive
Madison, WI 53711 USA

Toll Free in US (877) 320-6688 / (608) 310-5100 T
(608) 310-5101 F
fcdi-support@fujifilm.com E
www.fujifilmcdi.com W

Certificate of Analysis

**Product Name:**	iCell® Astrocytes 2.0	**Lot Number:**	107362
**Catalog Number:**	C1249	**Unit Size**	≥1x10^6 cells/vial
**Donor ID:**	01279	**Use By:**	12 months from date of shipment
		**Storage Conditions:**	Vapor Phase LN_2

This product is subject to use restrictions which can be found in FUJIFILM Cellular Dynamics, Inc. Standard Terms and Conditions accessible at https://fujifilmcdi.com/assets/tnc/standard.pdf. Failure to adhere to these use restrictions, among other things, will void the limited warranty applicable to this product.

For further assistance, contact technical support (fcdi-support@fujifilm.com).

Test Parameter	Result
**Viable Cells**	1.4x10^6 cells by trypan blue exclusion
**Purity, 7 days post thaw**	>99% $CD44^+$
	92% $CD49f^+$
	91% $GFAP^+$
	2% DCX^+
**Viability**	77% at thaw, by trypan blue exclusion
**Identity Confirmation**	Match by SNP genotyping
**Mycoplasma**	Pass by PCR
**Sterility**	Pass following USP71 Modified
**Glutamate Uptake**	Pass 7 days post-thaw

iPSC Line Authentication Statement

All FUJIFILM Cellular Dynamics, Inc. (FCDI) products are manufactured from tissue-derived induced pluripotent stem cell (iPSC) lines that were produced by FCDI. Each tissue sample received by FCDI (an Original Sample) is assigned a unique 5-digit identification code (ID). FCDI uses SNP analysis on genomic DNA (gDNA) isolated from the Original Sample to establish a DNA fingerprint for each ID.

The iPSC line and iPSC-derived differentiated cell type produced are identified by the Original Sample's ID and DNA fingerprinted to confirm identity with that Original Sample.

In the case a gene-edited iPSC is produced from a particular Original Sample, it is identified by the Original Sample's ID and confirmed using the DNA fingerprint. The gene-editing of the iPSC is confirmed using sequence analysis of the gene-edited region.

This lot passes all quality control specifications.

Quality Assurance Approval: Alison J Bahena Date: 07Aug2024

For Research Use Only. Not for use as a therapeutic or diagnostic. TEMP-00034.01 Effective Date:30 Sep 2019
© 2019 FUJIFILM Cellular Dynamics, Inc.
