# Supplementary material for: Static or microfluidic? A comparative pilot study of an in vivo-in vitro validated human iPSC-derived blood-brain barrier model for neurotoxicity hazard assessment
Source: NAM J. 2026 Oct 8;2:100142. doi: 10.1016/j.namjnl.2026.100142 (PMC13683853; doi:10.1016/j.namjnl.2026.100142)
Supplement: Supplementary file 2 [file mmc1.docx]

**Supplementary Data File 1. Certificates of analysis for hiPSC‑derived brain microvascular endothelial cells (BMECs), pericytes and astrocytes**

The certificates of analysis (CoA) display the identity of cell types in the tricellular blood‑brain barrier (BBB) model include expression cell‑specific markers, which were analyzed by flow cytometry. BMECs express multiple key transporter proteins such as permeability glycoprotein 1 (PGP, MDR1), glucose transporter 1 (GLUT1), CD98 (SLC3A2) and transferrin receptor protein 1 (TFRC/TfR1). Thereby, major BMEC transport mechanisms in terms of active efflux (PGP), carrier‑mediated transport (GLUT1 and CD98) and receptor‑mediated transcytosis (TFRC) are represented. Identification of pericytes included the expression of two brain pericyte markers in terms of platelet-derived growth factor receptor beta (PDGFRβ) and neural/glial antigen 2 (NG2). Astrocytes expressed the astrocyte‑specific marker glial fibrillary acidic protein (GFAP).
