# Supplementary material for: Static or microfluidic? A comparative pilot study of an in vivo-in vitro validated human iPSC-derived blood-brain barrier model for neurotoxicity hazard assessment
Source: NAM J. 2026 Oct 8;2:100142. doi: 10.1016/j.namjnl.2026.100142 (PMC13683853; doi:10.1016/j.namjnl.2026.100142)
Supplement: Supplementary file 3 [file mmc2.docx]

**Static or microfluidic? A comparative pilot study of an *in vivo‑in vitro* validated human iPSC‑derived blood‑brain barrier model for neurotoxicity hazard assessment**

**– Supplementary data file 2**

**Table 1. Compound list**

| **Compound** | **Supplier** | **Catalogue No.** | **CAS No.** | **Purity [%]** | | **Solvent** |
| --- | --- | --- | --- | --- | --- | --- |
| Sodium fluorescein | Sigma | F6377 | 518‑47‑8 | na | Medium | |
| Loperamide | Sigma | L0750000 | 34552‑83‑5 | > 99 | Methanol | |
| Verapamil | Sigma | V4629 | 152‑11‑4 | > 99 | Methanol | |
| Verapamil‑d_3_ | MCE | HY‑A0064S | 2714485‑49‑9 | > 99 | Methanol | |
| Cyclosporin A | Sigma | C‑093 | 59865‑13‑3 | > 95 | Acetonitrile | |
| Flumazenil | Sigma | 5.05991 | 1912‑24‑9 | > 98 | DMSO | |
| Flumazenil‑d_5_ | TRC | F500452 | 78755‑81‑4 | > 95 | DMSO | |

**Sample analysis**

Loperamide, Verapamil and Flumazenil were analyzed using a Vanquish LC system coupled to an Orbitrap Exploris 480 mass spectrometer (Thermo Fisher Scientific, Bremen, Germany). Chromatographic separation used an EC 150/2 Nucleodur C18 column (1.8 μm, 2.0 × 150 mm) (Macherey-Nagel, Düren, Germany, 760075.20). At flow rate 0.3 mL/min the following 13 min‑gradient of solvent A (H_2_O with 0.1 % formic acid) and B (acetonitrile with 0.1 % formic acid) was used: 0.5 min 5 % solvent B, increasing over 9.5 min to 100 % B, steady 100 % B for 3 min and finally 1 min with 5 % B. In the MS, ionization was achieved using a heated electrospray ion (HESI) source in positive (3.5 kV) and negative (3.0 kV) ion mode. The ion transfer tube and vaporizer temperature were kept at 300 °C. The precursor ion isolation window was set to 1 *m*/*z*. Overall, seven experiments were performed, including two full scans with an Orbitrap resolution of 120 000 FWHM at *m/z* 200 over a scan range of 70 *m*/*z* to 700 *m*/*z* in positive and negative ionization mode and five MS^2^ experiments in positive ionization mode with a resolution of 30 000 FWHM at *m/z* 200.
